# Attention-deficit hyperactivity disorder traits are a more important predictor of internalising problems than autistic traits

**DOI:** 10.1038/s41598-022-26350-4

**Published:** 2023-01-16

**Authors:** Luca D. Hargitai, Lucy A. Livingston, Lucy H. Waldren, Ross Robinson, Christopher Jarrold, Punit Shah

**Affiliations:** 1 Department of Psychology, University of Bath, Bath, BA2 7AY UK; 2 Neuroscience and Mental Health Research Institute, Cardiff University, Cardiff, UK; 3 Institute of Psychiatry, Psychology and Neuroscience, King’s College London, London, UK; 4 School of Psychological Science, University of Bristol, Bristol, BS8 1TU UK

**Keywords:** Psychology, Human behaviour

## Abstract

Autism Spectrum Disorder (ASD) and Attention-Deficit Hyperactivity Disorder (ADHD) are both linked to internalising problems like anxiety and depression. ASD and ADHD also often co-occur, making their individual statistical contributions to internalising disorders difficult to investigate. To address this issue, we explored the unique associations of self-reported ASD traits and ADHD traits with internalising problems using a large general population sample of adults from the United Kingdom (*N* = 504, 49% male). Classical regression analyses indicated that both ASD traits and ADHD traits were uniquely associated with internalising problems. Dominance and Bayesian analyses confirmed that ADHD traits were a stronger, more important predictor of internalising problems. However, brief depression and anxiety measures may not provide a comprehensive index of internalising problems. Additionally, we focused on recruiting a sample that was representative of the UK population according to age and sex, but not ethnicity, a variable that may be linked to internalising disorders. Nevertheless, our findings indicate that while ASD and ADHD uniquely predict internalising problems, ADHD traits are a more important statistical predictor than ASD traits. We discuss potential mechanisms underlying this pattern of results and the implications for research and clinical practice concerning neurodevelopmental conditions.

## Introduction

Autism Spectrum Disorder (ASD) is characterised by social-communication difficulties and restrictive and repetitive patterns of behaviour^1^. In addition to core ASD symptoms, a large body of research has focused on the associations between ASD and mental health problems, particularly internalising disorders. For example, recent meta-analyses have indicated that anxiety disorders, depression and obsessive–compulsive disorder (OCD) are more prevalent in autism compared to the general population^2,3^. Notably, internalising problems have also been linked to lower quality of life, physical wellbeing, and social functioning in ASD^4–7^, making them important targets for intervention. Attention-deficit hyperactivity disorder (ADHD)—characterised by inattention and/or hyperactivity and impulsivity^1^—is another neurodevelopmental condition associated with internalising disorders, including anxiety, depression and OCD^8–10^. Research has further indicated that co-occurring depression and anxiety is associated with greater social difficulties in people with ADHD, which can often be difficult to differentiate from ASD symptomology^11,12^. Moreover, anxiety and depression are thought to underlie the relationship between childhood ADHD and lower quality of life in adulthood^13^.

It is now clear that both ASD and ADHD are associated with internalising problems, which also contribute to many other health difficulties (e.g., sleep disorders;^14^) and a lower quality of life in people with these neurodevelopmental conditions^15^. Surprisingly, however, there is far greater emphasis on ASD over ADHD in both research on internalising problems^16^ and clinical practice^17^. To complicate matters, ASD and ADHD often co-occur, with a recent estimate that around 28% of autistic individuals have co-occurring ADHD^3^. The diagnostic co-occurrence and overlapping cognitive profiles between ASD and ADHD (e.g., emotional and attentional atypicalities^18^) makes it especially difficult to statistically examine their unique, relative contributions to internalising problems. There is little empirical research on this topic and almost nothing in adults, despite indications that the prevalence of anxiety and depression increases notably in adulthood in these clinical populations^19–22^. Existing studies, mainly in children and adolescents, have used small samples and/or seldom report effect sizes [e.g.,^23–25^]. They are likely to be under-powered and it is difficult to derive implications for clinical practice based on such research [see also,^15^].

A trait-based approach to the investigation of neurodevelopmental phenomena may be a useful way to overcome these limitations in previous research, given that both ASD and ADHD are dimensionally distributed across the general population [e.g.,^26–28^]. Instead of using diagnostic data, this involves using large, non-clinical samples of participants who complete self-report measures of ASD [e.g.,^29^] and ADHD [e.g.,^30^] traits. Harnessing the strength of this approach, we aimed to quantify the relative contributions of ASD and ADHD to anxiety and depression symptoms. Following the previous literature, we expected that both ASD and ADHD traits would be associated with these internalising problems. Critically, the present study focused on using a series of statistical methods to establish whether ASD or ADHD traits were the stronger and more important predictor of internalising problems.

## Methods

Recruitment through *Prolific* obtained a large general population sample of adults, representative of the United Kingdom in terms of age and sex distributions (based on Office for National Statistics data^31^). The sample had 504 participants (247 [49%] male; 257 [51%] female), aged 18–79 years (*M* = 45.03, *SD* = 15.41). This gave us at least 95% statistical power to detect small-to-medium unique effects in the multivariate analyses, where *f*^2^ = 0.05 and *α* = 0.05 (2-tailed;^32^). Participants provided their age (years), sex, and education level between 0 (no qualifications) and 7 (PhD or equivalent) on the International Standard Classification of Education^33^. No additional information was collected about clinical diagnoses. Participants completed the following self-report measures in a randomised order.

The 28-item Short Autism-Spectrum Quotient (AQ-Short;^29^) assessed social and non-social ASD traits, generating a score between 28 (few autistic traits) and 112 (many autistic traits). Importantly, the AQ-Short is suitable for use in both males and females as it is potentially less biased towards the male autism phenotype compared to other ASD trait measures^34^. The AQ-Short had good reliability in the present study (*α* = 0.84; *ω* = 0.83).

The Adult ADHD Self-Report Scale (ASRS^30^) assessed ADHD traits. Its 18 items measure the frequency of symptoms related to inattention and hyperactivity/impulsivity, producing scores between 0 (no ADHD traits) and 72 (many ADHD traits). Comparing the ASRS to clinician ratings, Kessler and colleagues^30^ reported that the classification accuracy of the ASRS for ADHD was 96.2%, supporting its construct validity. In the present study, the ASRS had good reliability (*α* = 0.90; *ω* = 0.90).

The 7-Item Generalised Anxiety Disorder Scale (GAD-7^35^) assessed how frequently participants experienced anxiety symptoms in the preceding 2-week period, leading to a score between 0 (no anxiety) and 21 (severe anxiety). Spitzer et al.^35^ showed that the GAD-7 had good convergent validity using the Beck Anxiety Inventory (*r* = 0.72) and the Symptom Checklist-90 (*r* = 0.74). The GAD-7 had good reliability in the present study (*α* = 0.93; *ω* = 0.93).

The 9-Item Depression Module of the Patient Health Questionnaire (PHQ-9^36^) measured the frequency of depression symptoms in the preceding 2-week period, generating a score between 0 (no depression) and 27 (severe depression). Martin et al.^37^ showed that the PHQ-9 was associated with the Brief Beck Depression Inventory (*r* = 0.73), demonstrating its convergent validity and, in the present study, this measure had good reliability (*α* = 0.92; *ω* = 0.92).

### Ethics approval and consent to participate

Ethical clearance was granted by a University of Bath Ethics Committee. All procedures performed in studies involving human participants were in accordance with the ethical standards of the institutional committee and with the 1964 Helsinki Declaration and its later amendments or comparable ethical standards. Participants gave informed consent prior to participation and were debriefed following completion.

## Results

The data were first analysed using JASP (Version 0.14.1)^38^. Based on predetermined criteria (i.e., a Studentized Residual with an absolute value ≥ 3 and a Cook’s Distance ≥ 0.5), no datapoints were identified as multivariate outliers. Correlations among variables were assessed using two-tailed Pearson’s correlations, before conducting a multiple regression analysis to investigate the associations of ASD traits and ADHD traits with internalising disorder symptoms, controlling for sex, age, and education level.

While multicollinearity was not a concern (all VIF values < 1.19), the visual inspection of a Q-Q plot of the standardised residuals and a scatterplot of the standardised residuals against the standardised predicted values indicated that the assumptions of normality and homoscedasticity were potentially violated. Thus, 95% bias-corrected and accelerated bootstrap confidence intervals (2000 resamples) are reported to complement the ordinary least squares estimates.

To determine the relative statistical importance of each predictor variable, dominance analyses were then conducted using the *yhat* package^39^ in RStudio (Version 1.3.959)^40^. Finally, two Bayesian regression analyses were carried out in JASP to derive Bayes Factors and thus quantify the evidence supporting ASD and ADHD traits as unique predictors of internalising problems.

Descriptive statistics and correlations are presented in Table 1. In line with the expected overlap between ASD and ADHD, moderate correlations were observed between ASD and ADHD traits, *r* = 0.30, *p* < 0.001. Both ASD and ADHD traits were correlated with greater anxiety (ASD: *r* = 0.28, *p* < 0.001; ADHD: *r* = 0.56, *p* < 0.001) and depression (ASD: *r* = 0.33, *p* < 0.001; ADHD: *r* = 0.55, *p* < 0.001). Anxiety and depression were also highly correlated (*r* = 0.81, *p* < 0.001). Therefore, to avoid concerns with multicollinearity in the multivariate analyses, anxiety and depression were combined into a composite measure of internalising problems [following^41,42^]. Both ASD traits, *r* = 0.32, and ADHD traits, *r* = 0.58, were correlated with greater internalising problems (all *ps* < 0.001).

**Table 1:** Descriptive Statistics and Pearson’s Correlations.

Measures	Mean (SD)	1	2	3	4	5	6	7
1. ASD Traits	65.69 (11.02)	–						
2. ADHD Traits	26.10 (11.46)	.30***	_					
3. Age (years)	45.03 (15.41)	− .04	− .23***	_				
4. Sex (0 = Female, 1 = Male)	–	.13**	− .09	.01	_			
5. Education Level	–	− .09*	− .03	− .11*	.03	_		
6. Anxiety Symptoms	5.92 (5.40)	.28***	.56***	− .27***	− .09*	− .04	_	
7. Depression Symptoms	7.14 (6.34)	.33***	.55***	− .27***	− .01	− .07	.81***	_
8. Internalising Problems	0.00 (0.95)	.32***	.58***	− .28***	− .05	− .06	.95***	.95***

*p < .05, **p < .01, ***p < .001.

Multiple regression (Table 2) assessed the unique associations of ASD and ADHD traits with internalising problems, including age, sex, and education level as covariates. Both trait ASD, *β* = 0.17, *p* < 0.001, and ADHD, *β* = 0.49, *p* < 0.001, were unique predictors of internalising problems. Inspection of beta coefficients suggested that trait ADHD was a stronger predictor than trait ASD. Comparing beta coefficients, however, is not sufficient to test the relative importance of predictors [see^43^]. Therefore, using dominance analysis, as increasingly used in autism research [e.g.,^44,45^], we calculated General Dominance Weights (GDWs), where larger weights indicated greater variance explained in internalising problems. These GDWs were used to rank the predictors in order of statistical importance [following^46^]. ADHD traits (GDW = 0.27) clearly dominated ASD traits (GDW = 0.06) as the most important predictor of internalising problems (Table 2). The analysis also revealed that this dominance relationship, estimated via bootstrapping (2000 resamples; following^47^), had a reproducibility rate of 100%. This indicated that ADHD traits would always dominate ASD traits as a predictor of internalising problems at the population level.

**Table 2:** Regression, Dominance, and Bayesian Analyses.

Predictor	*B* [95% CIs]	*SE(B)*	*β*	*p*	*sr*^*2*^	GDW	BF_10_
ASD traits	0.01 [0.01, 0.02]	0.003	.17	< .001	.024	.063	2160.73
ADHD traits	0.04 [0.03, 0.05]	0.003	.49	< .001	.201	.268	1.049 × 10^29^
Age	− 0.01 [− 0.02, − 0.01]	0.002	− .17	< .001	.027	.053	–
Sex	− 0.06 [− 0.19, 0.08]	0.068	− .03	.390	.001	.003	–
Education level	− 0.02 [− 0.06, 0.01]	0.018	− .05	.193	.002	.003	–
Overall Model Fit	*F* (5, 498) = 63.56, *p* < .001, *R*^*2*^ = 0.39						

95% bias − corrected and accelerated bootstrap confidence intervals for *B* using 2000 resamples. General Dominance Weight (GDW, with higher values indicating a more important predictor). Sex is coded as 0 = female; 1 = male.

Finally, two Bayesian analyses were conducted, as recommended alongside traditional frequentist statistics [see^48^]. We compared models containing the predictor of interest (either ASD traits or ADHD traits) with null models including all other variables, following recent autism research [e.g.,^49^]. The Bayes Factors (BF_10_), therefore, indexed ASD and ADHD traits as unique predictors of internalising problems. There was “*extreme*” evidence that both ASD and ADHD traits predicted internalising problems but, consistent with the foregoing analyses, ADHD traits, BF_10_ = 1.049 × 10^29^, were a much stronger predictor of internalising problems than ASD traits, BF_10_ = 2160.73. The overall pattern of results was identical to separate analyses for anxiety (Table 3) and depression (Table 4) symptoms.

**Table 3:** Multiple Regression, Dominance and Bayesian Analyses for Anxiety.

Predictor	*B* [95% CIs]	*SE(B)*	*β*	*p*	*sr*^*2*^	*GDW*	*BF*_*10*_
ASD traits	0.07 [0.03, 0.11]	0.019	.14	< .001	.017	.048	76.611
ADHD traits	0.22 [0.19, 0.26]	0.018	.47	< .001	.189	.250	3.668 × 10^26^
Age	− 0.06 [− 0.08, − 0.03]	0.013	− .16	< .001	.024	.048	–
Sex	− 0.71 [− 1.54, 0.01]	0.396	− .07	.072	.004	.007	–
Education level	− 0.08 [− 0.28, 0.12]	0.103	− .03	.439	.001	.001	–
Overall Model Fit	*F* (5, 498) = 54.60, *p* < .001, *R*^*2*^ = 0.35						

95% bias-corrected and accelerated bootstrap confidence intervals for *B* using 2000 resamples. General Dominance Weight (GDW, with higher values indicating a more important predictor). Sex is coded as 0 = female; 1 = male.

**Table 4:** Multiple Regression, Dominance, and Bayesian Analyses for Depression*.*

Predictor	*B* [95% CIs]	*SE(B)*	*β*	*p*	*sr*^*2*^	*GDW*	*BF*_*10*_
ASD traits	0.10 [0.05, 0.15]	0.022	.18	< .001	.027	.066	4859.401
ADHD traits	0.25 [0.21, 0.30]	0.022	.45	< .001	.174	.235	4.807 × 10^24^
Age	− 0.07 [− 0.10, − 0.04]	0.015	− .17	< .001	.025	.048	–
Sex	0.10 [− 0.82, 0.97]	0.465	.01	.831	.000	.001	–
Education level	− 0.20 [− 0.44, 0.04]	0.121	− .06	.102	.003	.004	–
Overall Model Fit	*F* (5, 498) = 54.74, *p* < .001, *R*^*2*^ = 0.35						

95% bias-corrected and accelerated bootstrap confidence intervals for *B* using 2000 resamples. General Dominance Weight (GDW, with higher values indicating a more important predictor). Sex is coded as 0 = female; 1 = male.

## Discussion

We determined the relative contributions of ASD and ADHD to internalising problems using a trait-based approach in a large general population sample. Consistent with the previous literature [e.g.,^2,8^], both ASD and ADHD traits predicted greater internalising problems. The regression analyses also indicated that ADHD traits may be a stronger predictor of internalising problems than ASD traits. This was formally confirmed with dominance analyses, which showed that ADHD traits dominated ASD traits in predicting internalising problems, with reproducibility estimates highlighting that this finding is almost certain to exist in the general population. Bolstering these results, Bayes Factors made it even clearer that ADHD traits were more predictive of internalising problems than ASD traits. Overall, we report converging evidence that ADHD traits are a stronger and more important predictor of internalising problems than ASD traits.

To our knowledge, this is the first evidence that ADHD traits are more predictive of internalising problems than ASD traits. While its novelty might appear surprising, it is reflective of the dearth of research on this topic. Unfortunately, ASD has often been prioritised over ADHD in both research on internalising problems and clinical practice, particularly for anxiety [see^16,17^]. This has led to major gaps in the literature, which this study has helped to address. Based on the robustness and consistency of our results, we argue that greater emphasis should be placed on the role of ADHD in mental health research and clinical practice on neurodevelopmental conditions. Our finding that ADHD traits dominated ASD traits in predicting internalising disorder symptoms could inform strategies to identify individuals who are at an increased risk of internalising problems. This might allow preventative measures and interventions to be implemented at an earlier age, which, for example, could focus on managing ADHD symptoms for a greater impact on ameliorating internalising problems and improving mental wellbeing in adults. Developmental, longitudinal research will now be required to advance this line of work, for example, to determine the direction of the relationships between neurodevelopmental and internalising symptoms across development. Equally, addressing other gaps in the literature, this study offers novel insight into the link between neurodevelopmental conditions and mental health in adults, a population that is often understudied in the context of ASD and ADHD^15^. Our findings suggest that the management of ADHD traits in adults, with or without ASD, has potential to reduce internalising problems, which could supplement clinical interventions directly targeting anxiety and depression (e.g., anti-depressant medications, talking therapies), although replication is, of course, now required in clinically diagnosed samples. Given that ADHD/ASD traits share similar correlates with clinical diagnoses [see^27^], we hypothesise that a similar pattern of results will be found in such research. Indeed, a recent study in clinically diagnosed autistic people found that ADHD traits, compared to autistic traits, were more strongly associated with a lower quality of life^50^. As quality of life and internalising problems are correlated^51,52^, we would expect that our pattern of results will, in future, be found in a sample of clinically diagnosed autistic people.

Before these potential suggestions could be translated into practice, there is a need to investigate the mechanisms underlying our results. While the specific neurocognitive processes that underpin our findings are unknown, a possible explanation for this pattern of results may be the differential executive function difficulties that characterise ASD and ADHD. A systematic review by Craig and colleagues^53^ revealed that, while both neurodevelopmental conditions are associated with executive dysfunction, autistic individuals tend to have more problems with cognitive flexibility and planning, while those with ADHD have more response inhibition difficulties [see also^54^]. Crucially, such response inhibition problems have also been documented in depression^55–57^ and anxiety^58–60^. We therefore speculate that response inhibition difficulties, which appear to be a cognitive feature of ADHD, may also potentially underpin the stronger association between ADHD traits and internalising problems. The residual link between autism and internalising problems may be driven by other atypicalities often reported in these conditions, such as alexithymia [see^61^]. Our study was neither designed nor able to test for such mechanisms, and alexithymia has not been investigated in ADHD controlling for ASD, however it provides some direction for future research on the processes underlying differential contributions of ADHD and ASD to poor mental health.

In considering mechanisms to explain our findings, it may also be important to think about the overlap between ADHD, ASD and internalising problems at the genetic level [see^15^ for a review]. Family studies have consistently shown an increased prevalence of depression in biological relatives of individuals with ADHD and/or ASD [e.g.,^62,63^] with similar findings for anxiety [e.g.,^64,65^]. Further, studies identifying common genetic variants (i.e., via genome-wide association studies; GWAS) demonstrate genetic correlations between internalising disorders and both ADHD and ASD [e.g.,^66,67^]. Interestingly, a recent GWAS of eight major psychiatric conditions^68^ showed that, whilst ADHD and ASD show similar genetic correlations with depression (0.44 and 0.45, respectively), significant genetic correlations with anxiety were found with ADHD, but not ASD. That is, a pattern of results in a genetic study in line with our findings. Future research could investigate, more directly, whether genetic factors can explain why ADHD traits are more strongly associated with internalising problems than autistic traits.

Further research is also required to address limitations of the present study. First, internalising problems were assessed using brief depression and anxiety measures, which may not provide a comprehensive index of internalising problems. Additionally, several internalising symptoms measured by these brief screeners (e.g., insomnia, restlessness and changes in appetite) overlap with the symptom profiles of ASD and ADHD^15^, suggesting that some participants may have been scoring on certain items due to their neurodevelopmental traits rather than experiences of anxiety and/or depression. A future replication of our study using a more in-depth measure that overlaps less with items on the ASRS and AQ-Short (e.g., the Depression, Anxiety and Stress Scales;^69^), may be helpful in testing the validity and replicability of our findings. Second, ethnicity was not considered as we focused on recruiting a sample that was UK representative by age and sex alone. However, given that there may be a link between ethnicity and internalising problems [e.g.,^70,71^], it will be important to assess whether the dominance relationship observed holds after accounting for participants’ ethnicity and other socio-demographic factors. Finally, while the trait-based approach was an important step in understanding the unique contributions of ASD and ADHD to internalising problems, the study will require a conceptual replication with clinical samples. Such research would benefit from using measures of internalising symptoms that have been validated in this population, which are currently lacking^15^. This will be difficult to achieve as it is only with the most recent revision of the Diagnostic and Statistical Manual of Mental Disorders^1^ that individuals can receive concurrent ASD and ADHD diagnoses^72^. Consequently, recruiting a sufficiently large sample of clinically diagnosed participants, as required to undertake the analyses we have employed, will require considerable resources. Our study hopefully provides the impetus for such research.

Overall, our findings demonstrate that ASD and ADHD uniquely predict internalising problems, such as anxiety and depression. Crucially, ADHD traits were a much more important predictor of internalising problems and our analyses indicated that this relationship is almost certain to occur in the overall population. While further research is necessary to replicate these findings and elucidate mechanisms that underpin the observed relationships, our study provides important new evidence linking neurodevelopmental conditions and mental health in adulthood.

## Data Availability

The anonymised data from the present study are available from the corresponding author on request.

## Abbreviations

ADHD: Attention-Deficit Hyperactivity Disorder
AQ-Short: 28-item Short Autism-Spectrum Quotient
ASD: Autism Spectrum Disorder
ASRS: Adult ADHD Self-Report Scale
BF_10_: Bayes Factor
GAD-7: 7-Item Generalised Anxiety Disorder Scale
GDW: General Dominance Weight
OCD: Obsessive-Compulsive Disorder
PHQ-9: 9-Item Depression Module of the Patient Health Questionnaire.
